# Impact of gamma rays on the *Phaffia rhodozyma* genome revealed by RAPD-PCR

**Published:** 2011-12

**Authors:** N Najafi, Ramin Hosseini, AR Ahmadi

**Affiliations:** 1 Women Research Centre, Biomedical Department, Alzahra University, Tehran, Iran; 2 Agricultural Biotechnology Department, Faculty of Engineering, Imam Khomeini International University, Qazvin, Iran

**Keywords:** astaxanthin, *Phaffia rhodozyma*, RAPD, UPGMA

## Abstract

**Background and Objectives:**

*Phaffia rhodozyma* is a red yeast which produces astaxanthin as the major carotenoid pigment. Astaxanthin is thought to reduce the incidence of cancer and degenerative diseases in man. It also enhances the immune response and acts as a free-radical quencher, a precursor of vitamin A, or a pigment involved in the visual attraction of animals as mating partners. The impact of gamma irradiation was studied on the *Phaffia rhodozyma* genome.

**Materials and Methods:**

Ten mutant strains, designated Gam1-Gam10, were obtained using gamma irradiation. Ten decamer random amplified polymorphic DNA (RAPD) primers were employed to assess genetic changes.

**Results:**

Nine primers revealed scorable polymorphisms and a total of 95 band positions were scored; amongst which 38 bands (37.5%) were polymorphic. Primer F with 3 bands and primer J20 with 13 bands produced the lowest and the highest number of bands, respectively. Primer A16 produced the highest number of polymorphic bands (70% polymorphism) and primer F showed the lowest number of polymorphic bands (0% polymorphism). Genetic distances were calculated using Jaccard's coefficient and the UPGMA method. A dendrogram was created using SPSS (version 11.5) and the strains were clustered into four groups.

**Conclusion:**

RAPD markers could distinguish between the parental and the mutant strains of *P. rhodozyma*. RAPD technique showed that some changes had occurred in the genome of the mutated strains. This technique demonstrated the capability to differentiate between the parental and the mutant strains.

## INTRODUCTION

*Phaffia rhodozyma* is a red yeast which produces astaxanthin as the major carotenoid pigment. This compound enhances pigmentation of fish, crustaceans, and poultry (1). There is a lot of commercial interest towards astaxanthin production, not only as a pigmentation source but also as a potent anti-oxidative reagent that can delay aging in animals (2). One approach to increase a character of interest in an organism is mutagenesis. Mutagenesis with gamma rays has been induced and applied in a variety of organisms, including *Phaffia rhdozyma* (3, 4), *Hanesula anomala* and *Rhodotorula rubra* yeasts (5), *Jatropa curate*, *Chamaecyparis obtuse* Sieb. et Zucc (7), a shrub with significant economic importance (6), *Aspergillus niger* (8) and *Saccharomyces cervisiae* (9). The pattern and distribution of DNA fragmentation produced by linear energy transfer (LET) radiations such as gamma rays are characteristically different to those produced by high-LET radiations such as fast neutrons, in both prokaryotic and eukaryotic organisms. It has been proposed that low LET radiations produce a higher rate of single-stranded breaks (SSBS) in DNA than double-stranded breaks (DSBS), per unit dose, compared to high-LET radiations (10, 11). Gamma irradiation produces oxygen radicals generated by the hydrolysis of water (12) and could induce chromosomal rearrangements (13). A technique widely used for detecting alterations in DNA sequences induced by mutagenic agents such as gamma rays is randomly amplified polymorphic DNA (RAPD) based on polymerase chain reaction (PCR). This is a useful analysis tool for identifying polymorphisms in DNA sequences (5–9). The advantage of RAPD over other techniques is the low technical input and small quantity of DNA needed for the analysis (14, 15).

The aim of this study was to investigate and discover possible DNA alterations induced by gamma rays in the 10 mutant strains of *P. rhodozyma*. Genetic differences were analysed between 11 strains by RAPD analysis and based on the similarity indices, a dendrogram was constructed.

## MATERIALS AND METHODS

**Obtaining mutant strains and culture media.** The astaxanthin hyper-producing mutant JH-82 was derived from a wild type strain by mutagenesis with nitrosoguanidine (16) and selected for its dark orange color. Jh-82 was used as the parental type for the mutagenesis study. The yeasts were cultured on YMB medium (10 g/L glucose, 5 g/L bacto-peptone, 3 g/L yeast extract, 3 g/L malt extract) (Difco) at 20°C in a shaking incubator (140 rpm). When yeasts reached the log phase, they were irradiated at room temperature using ^60^CO gamma ray irradiator type GC-220 as a source with 1, 2, 3, 3.5, 4, 4.5, 5, 5.5, 6 and 7 kGy doses. The mutant strains obtained were as follows: Gam1, Gam 2, Gam 3, Gam 4, Gam 5, Gam 6, Gam 7, Gam 8, Gam 9 and Gam 10.

**Preparation of serial dilution of the mutant cells.** Following the irradiation, 10^0^–10^-8^ serial dilutions of the cells were prepared from all irradiated cells. Then 50 µl from each dilution was cultured on the solid YMB medium at 19°C. After 20 days, colonies appeared on the medium.

**Preparation of yeast DNA.** Yeast DNA was isolated using a quick procedure (17) with some modifications. Briefly, the cells were cultured overnight at 20°C in a 10 ml of YMB medium, at 140 rpm on a shaker. Cells were precipitated at 2000 *g* for 5 min. About 200 µl of lysis buffer containing 0.5 mM EDTA, 10% (w/v) SDS, 1 M Tris-HCl, 5 M NaCl and 0.02% (v/v) Triton X-100, 200 µl phenol: chloroform: isoamyl alcohol (1: 24: 25) and 0.3 g of acid washed glass beads were added to the pellet. The mixture was vortexed for 4 min and 200 µl TE (1 mM EDTA, 10 mM Tris-HCl, pH 8) was added and centrifuged at 7000×*g* for 5 min. The supernatant was transferred into a new tube. Then 1 ml 100% EtoH was added. The tube was centrifuged at 14500×*g* and the pellet was resuspended in 400 µl RNAase (10 µg/ml) and incubated at 37°C for 2 h. An aliquot of 10 µl ammonium acetate (4 M) and 1 ml of 100% EtoH were added. Tube was centrifuged at 14500×*g* for 1 min and pellet was washed with 1 ml of 70% EtoH and air dried. DNA was resuspended in 50 µl of TE, its purity and concentration were determined by measuring absorbance at 260 and 280nm wavelenghths. A260/A280 ratio was between 1.8-2.0 in all extracted DNA samples.

**RAPD amplification and analysis.** Random DNA amplifications using 10 decamer primers (Table 1) were performed in 20 µl reaction mixture containing: 1x PCR buffer (0.1% Triton X-100, 50 mM KCl, 10 mM Tris-Hcl pH 9.0 and 0.5 mM MgCl_2_), 100 ng of genomic DNA, 160 µM of dNTPs, 0.16 µM of primer (10 mer), 1.6 mM MgCl_2_ and 0.4U of Taq DNA polymerase (Cinnagen, Tehran, Iran). PCR was carried out in a PTC-1148 programmable thermal controller (BioRAD, USA). Reactions were run for 35 cycles with the following conditions: denaturation at 94°C for 30 s, annealing at 36°C for 45 s, and extension at 72°C for 1min and 45 s. An initial denaturation of 94°C for 4 min at and a final extension of 72°C for 10 min were set. Amplification products were run on a 1.5% agarose gel (w/v) in TBE buffer (0.89 M Tris-base pH 8.2, 0.89 M Boric acid, and 0.02 M EDTA). Gels were stained in ethidium bromide (0.5 µg/ml), visualized and photographed in a gel doc system (UVP, UK). Reproducibility of RAPD-PCR patterns was assessed by the repetition of RAPD analyses three times.

**Table 1:** The sequence of the 10 decamer RAPD primers, with the total number of amplified DNA fragments and the number of polymorphic fragments obtained by each primer.

Primer	Sequence 5′ to 3′	Amplified fragments	Polymorphic fragments
D	TGGCGTCGCT 9	9	6
F	CCCACTGACG 3	3	0
H	GGTCAACCCT 9	9	4
A16	AGCCACGGAA 10	10	7
C08	TGGACCGGTG 12	12	4
E07	AGATGCAGCC 7	7	2
J20	AAGCGGCCTC 13	13	2
P06	GTGGGCTGAC 8	8	2
P14	CCAGACGAAC 12	12	6
U19	GTCAGTGCGG 12	12	5

**Statistical analysis.** Only strong and clearly reproducible (between replicates) polymorphic DNA (RAPD) bands were scored as present (1) or absent (0) for each primer. Pairwise comparisons of the strains, based on the presence or absence of unique and shared bands, were used to generate similarity coefficient of Jaccard. The results were then analysed using the unweighted pair-group method with arithmetic average (UPGMA). Clustering the strains was carried out using SPSS version 11.5, SAHN (sequential agglomerative hierarchical) and nested clustering programs.

## RESULTS AND DISCUSSION

**Fig. 1:**
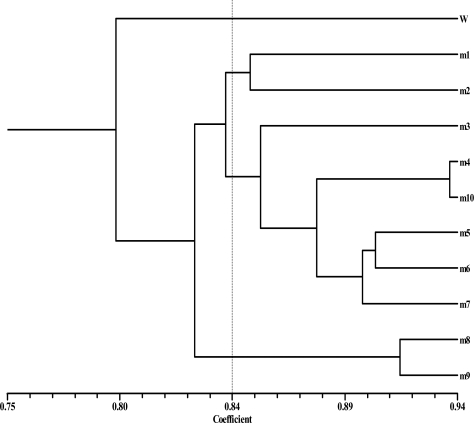
Dendrogram showing relationships between the 11 yeast strains of *Phaffia rhodozyma*. W: JH-82, m1-m10: Gam 1–Gam 10 mutant strains, respectively.

RAPD technique is shown to be able to detect mutations (5, 6, 18, 19) and genetic variation at intraspecific level (20) even though the most common application of RAPD has been to determine taxonomic variation and genetic mapping (6). It is suggested that with proper optimization, RAPD could be a sensitive and reliable technique to detect a wide range of DNA damage (19). In this study, different doses of gamma rays including 1, 2, 3 3.5, 4, 4.5 5, 5.5 6 and 7 kGy were applied on a *Phaffia rhodozyma* strain designated as JH-82 and 10 mutant strains (Gam 1– Gam 10) were obtained (Table 2). RAPD assay was employed to detect genomic alterations in the mutant strains and it could clearly show that some DNA changes had occurred in the mutant strains (Fig. 1).

**Table 2:** The gamma ray doses applied and the mutant strains that obtained from each dose.

Gamma dose	Mutant strain
1	Gam 6
2	---------
3	---------
3.5	Gam 3
4	----------
4.5	Gam 4
5	Gam 1, Gam 2, Gam 5
5.5	Gam 9, Gam 10
6	Gam 1
7	---------

**Fig. 2:**
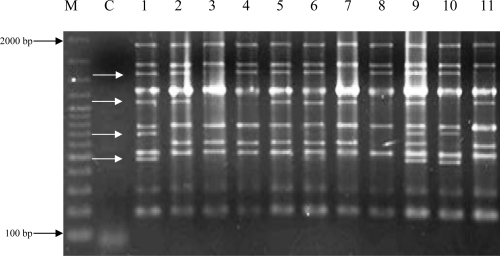
The amplified fragments of the 11 *Phaffia rhodozyma* strains, using U19 primer. C: negative control, lane 1: JH-82 strain, lanes 2–11 correspond to Gam 1 to Gam10, respectively. White arrows show some polymorphic amplified fragments between the parental and mutant strains.

The 10 decamer primers produced 95 bands amongst which 38 were polymorphic and 57 (62.5%) were monomorphic. The highest (13) and the lowest number of polymorphic bands (0) were produced by A16 and F primers, respectively (Table 1). The amplified DNA fragments ranged from 150 to 3,500 base pairs (bp) with different primers. A dendrogram was created based on the Jaccard's similarity coefficient and the mutant strains were clustered into four groups. Cophenetic correlation coefficient of the resulting dendrogram was 0.77. The parental type was classified in a separate group. Second group consisted of Gam 1 and Gam 2. Third group consisted of Gam 3, Gam 4, Gam 5, Gam 6, Gam 7 and Gam 10. Gam 8 and Gam 9 were placed in the last group. Based on the Jaccard's similarity coefficient, Gam 4 and Gam 10 showed the highest similarity with the similarity coefficient of 0.93 and JH-82 and Gam 9 showed the lowest similarity coefficient of 0.75 to each other (Fig. 1). Differences were apparent as the appearance of new amplified bands or the disappearance of some bands (Fig. 2). The disappearance of bands in the mutant strains may be related to such events as DNA damage (single or double strand breaks, modified bases site, oxidized bases, and bulky adducts), DNA-protein cross links, point mutations and complex chromosomal rearrangements induced by gamma irradiation (21). Irradiation is known as the best physical mutagen which dissociates water molecules and produces hydroxyl radicals and causes oxidative damage (21). The free radicals interact with biomolecules including DNA and scavenge electrons from them. This imposes damage to the structure and the activity of the DNA. When Taq polymerase encounters a damaged DNA, there could be a number of outcomes such as blockage, bypass and possible dissociation of the enzyme-DNA which will cause the loss of bands. However the appearance of new bands could be attributed to mutation rather than DNA damage (19, 22).

El-Sherebeny et al. (5) used gamma rays and fast neutrons to induce mutation in *Hansenula anomala* and *Rhodotorula rtubra* and employed RAPD assay to track genomic changes. They found fast neutrons more efficient in causing mutation than gamma rays. However, they reported that RAPD could detect changes in genetic make up of both yeasts. In our study, RAPD assay showed changes in number and presence or absence of some amplified bands between the studied strains. Some bands present in the parental strain disappeared in the mutants or vice versa (Fig. 2). These changes occurred in both large and small amplified fragments. This is in contrast to findings by Atienzar et al. (18). They reported that the disappearance of larger bands was more evident than that of shorter ones. Although genetic basis of the polymorphisms generated by RAPD is not well-defined, the RAPD assay proved useful a means of discovering genomic alterations induced by gamma rays in our study. As it has been reported by other studies in the past, this assay could be applied in mutation breeding studies as well.
